# Benefits of laparoscopic liver resection for liver tumors in obese patients: a meta-analysis

**DOI:** 10.3389/fonc.2024.1489261

**Published:** 2024-10-22

**Authors:** Jie Zhang, Cuifang Zeng, Rui Chen, Gang Tang, Rongxing Zhou

**Affiliations:** Division of Biliary Tract Surgery, Department of General Surgery, West China Hospital, Sichuan University, Chengdu, Sichuan, China

**Keywords:** laparoscopic liver resection, open liver resection, obesity, morbidity, meta-analysis

## Abstract

**Objective:**

The superiority of laparoscopic liver resection (LLR) and open liver resection (OLR) in obese patients remains controversial. The study aims to assess the available literature and compare the perioperative outcomes of LLR and OLR for liver tumors in obese patients.

**Methods:**

We searched PubMed, Cochrane Library, Embase, and Web of Science databases for studies comparing LLR and OLR. Odds ratios (ORs) and mean differences (MDs) with 95% confidence intervals (CIs) were calculated.

**Results:**

Nine studies were included, with a total of 1116 patients (LLR group: 482 patients; OLR group: 634 patients). Compared with OLR, LLR has lower overall morbidity (OR 0.47, 95% CI 0.34, 0.64), major complications (OR 0.45, 95% CI 0.25, 0.82), surgical site infection (OR 0.18, 95% CI 0.07, 0.48), bile leak (OR 0.45, 95% CI 0.22, 0.95), less blood loss (MD, -329.12 mL; 95% CI, -623.35, -34.88), and shorter length of stay (MD, -5.20 days; 95% CI, -7.43, -2.97). There were no significant differences in mortality, operation time, liver failure, and blood transfusion between the two groups.

**Conclusions:**

LLR for obese patients is safe and feasible. Compared to OLR, it offers better short-term outcomes. Further randomized controlled trials to verify the potential advantages of LLR over OLR are warranted.

## 1 Introduction

In recent years, with changes in lifestyle, the prevalence of obesity and its related diseases is rising worldwide (1, 2). Previous evidence has shown that obesity is associated with a variety of diseases, such as diabetes, hypertension, cardiovascular disease, and some types of malignancy (3, 4). A meta-analysis of 28 cohort studies involving 8,135,906 participants showed that elevated body mass index (BMI) was associated with the development of primary liver cancer (HR, 1.69; 95%CI 1.50-1.90), and the risk of primary liver cancer increased with increasing BMI (5). In addition, obesity also increases the degree of technical difficulty of surgery and is a risk factor for postoperative complications (6). Zimmitti et al. (7) found that obesity not only increased the operation time, but also increased the intraoperative blood loss and wound and respiratory related complications. How to improve the outcomes of obese patients after liver resection is a serious problem for surgeons.

Compared with traditional open surgery, laparoscopic surgery has the advantages of less damage to the abdominal wall, less intraoperative blood loss and faster postoperative recovery (8). Laparoscopic surgery has been widely used in liver resection. However, the superiority of laparoscopic liver resection (LLR) over open liver resection (OLR) in obese patients remains controversial. A retrospective study by Yoon et al. (9) showed that LLR can significantly reduce the blood loss, the incidence of complications, and the length of hospital stay. However, Yu et al. (10) found no significant differences in intraoperative blood loss, operation time, and postoperative hospital stay between LLR and OLR. To date, meta-analyses comparing the use of LLR versus OLR in obese patients are lacking.

Therefore, the objective of our meta-analysis was to compare perioperative outcomes between LLR and OLR for liver tumors in obese patients. These results may help provide evidence-based medical evidence for surgeons in selecting appropriate surgical approaches for obese patients.

## 2 Methods

### 2.1 Search strategy

This study was conducted in accordance with the Preferred Reporting Items for Systematic Reviews and Meta-Analyses (PRISMA) (11). The study protocol was registered with the PROSPERO database.

A systematic search using the EMBASE, Web of Science, PubMed, and Cochrane Library databases was conducted up to July 22, 2024. The detailed search strategy is presented in **Table 1**. In addition, we checked the reference lists of the identified articles and related reviews to further screen for eligible studies. No language restrictions were applied during the search process.

**Table 1:** Electronic search strategy.

Database	Search term (published up to June 29, 2024)	Number
PubMed	(Hepatectomy[Title/Abstract] OR liver resection[Title/Abstract]) AND (body mass index[Title/Abstract] OR BMI[Title/Abstract] OR obesity[Title/Abstract] OR obes*[Title/Abstract] OR overweight[Title/Abstract]) AND (laparoscopy[MeSH Terms] OR Laparoscop*[Title/Abstract])	171
Embase	(Hepatectomy OR liver resection).ab,kw,ti. AND (body mass index OR BMI OR obesity OR obes* OR overweight).ab,kw,ti. AND (laparoscopy or Laparoscop*).ab,kw,ti.	407
Cochrane Library Trials	((Hepatectomy OR liver resection):ti,ab,kw) AND ((body mass index OR BMI OR obesity OR obes* OR overweight):ti,ab,kw) AND ((laparoscopy OR Laparoscop*):ti,ab,kw)	49
Web of Science	(TS=(Hepatectomy OR liver resection)) AND (TS=(laparoscopy OR Laparoscop*)) AND TS=(body mass index OR BMI OR obesity OR obes* OR overweight)	338

### 2.2 Study selection

Studies included in this meta-analysis were chosen according to the PICOS (Patient, Intervention, Comparison, Outcomes, Study Type) criteria, as shown below: (1) Patient: Obese patients undergoing liver resection for liver tumors. Obesity is defined as BMI ≥30 (for Western studies) in accordance to the WHO classification or BMI ≥25 in accordance to the International Obesity Task Force (for Asian population) (2); (2) Intervention: LLR; (3) Comparison: OLR; (4) Outcomes: Primary outcomes encompassed mortality, overall morbidity and major complication. Secondary outcomes included blood loss, operation time, surgical site infection, liver failure, bile leak, blood transfusion, and length of stay; (5) Study type: randomized controlled trials (RCTs), cohort studies, and case-control studies.

The exclusion criteria were as follows: reviews, letters, single-arm studies, animal studies, case reports, editorials, conference abstracts, and repeated publications were excluded.

### 2.3 Data extraction

Data from all eligible studies were independently extracted by two authors, and any disagreements were resolved by discussion with a third-party author. The extracted data included author name, year of publication, study design, country, study population (sample size, age, diagnosis, and sex), and perioperative outcomes (mortality, morbidity, blood loss, operation time, blood transfusion, and length of stay). When data of interest were unavailable, the corresponding author was contacted to obtain the missing data.

### 2.4 Quality assessment

The quality assessment was conducted independently by two authors using the Newcastle-Ottawa Scale (NOS), which assigns a score on a 9-point scale. A score of ≥7 indicates high quality, and scores of 5–6 indicate moderate quality. Any discrepancies were resolved through discussion, with intervention by a third author whenever necessary.

### 2.5 Statistical analysis

Odds ratios (ORs) with corresponding 95% confidence intervals (CI) were calculated for qualitative variables and mean difference (MD) for quantitative data. The I² statistic was used to assess the degree of heterogeneity. A random-effects model was used if I² > 50%; otherwise, a fixed-effects model was employed (12). To explore the robustness of the results, we adopted the one-study exclusion method to evaluate the impact of each study on the pooled effect size. The meta-analysis was performed using the Review Manager software (version 5.3). Statistical significance was set at P-value of less than 0.05.

## 3 Results

### 3.1 Literature retrieval

**Figure 1:**
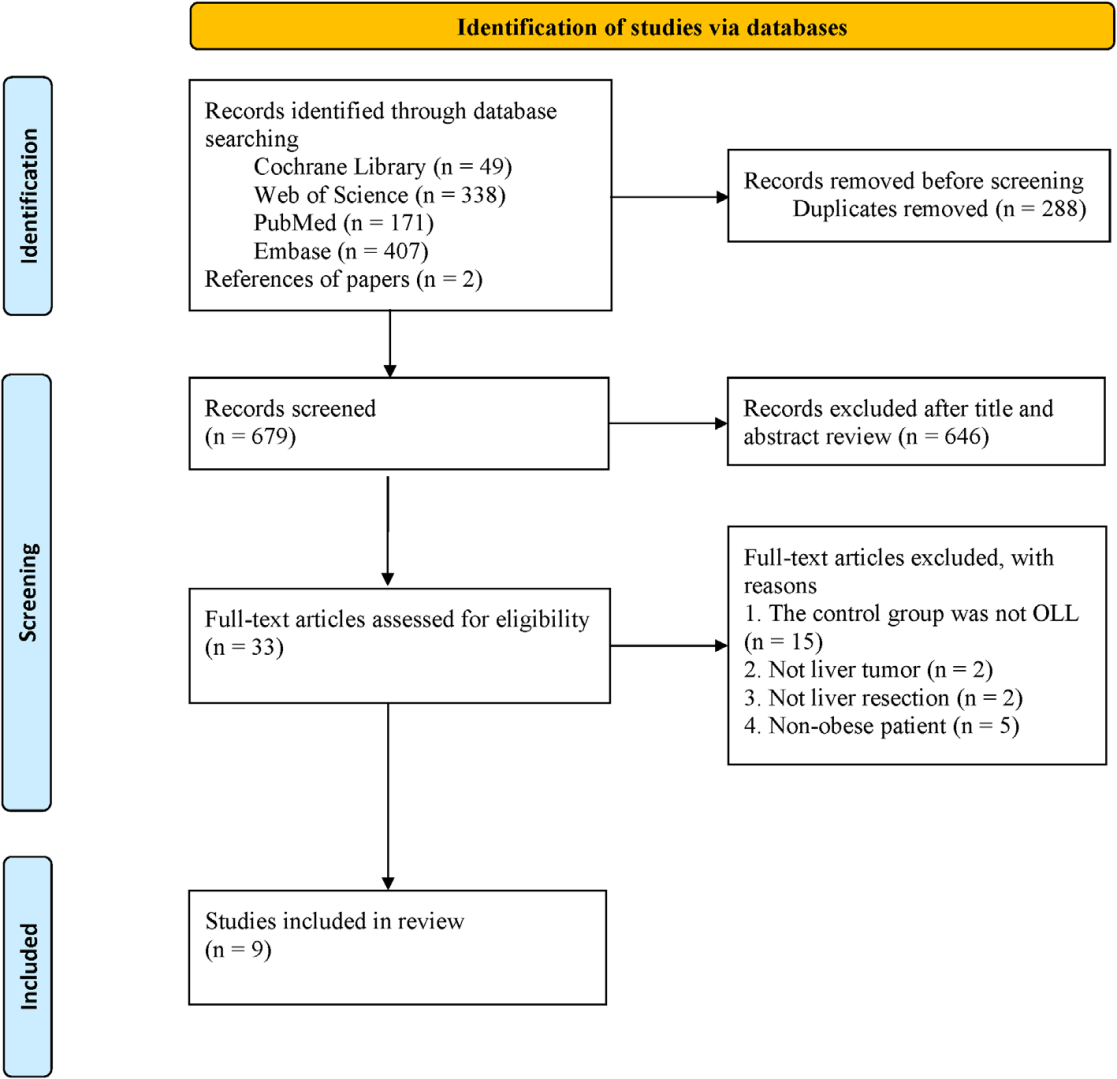
The PRISMA flowchart.

A total of 967 articles were retrieved from the database search. This included 288 duplicates, which were removed. After reviewing titles and abstracts, 646 papers were excluded, and the full texts of the remaining 33 studies were evaluated. Finally, 9 studies (1, 6, 9, 10, 13–17) were included in the final analysis (**Figure 1**).

### 3.2 Study characteristics and quality assessment

The main characteristics of the 9 included studies are summarized in **Table 2**. The studies were published between 2015 and 2024 and included 1116 patients (LLR group: 482 patients; OLR group: 634 patients). Indications for operative management were for malignancy only in 4 studies (6, 15–17), whilst benign and malignant disease processes were included in 5 studies (1, 9, 10, 13, 14). The included patients were mainly from Japan, Korea, Germany, and China. All studies (1, 6, 9, 10, 13–17) were considered of moderate to high quality, achieving a score of ≥6 based on the NOS (**Table 2**).

**Table 2:** Study Characteristics of the 9 included studies.

First author, year	Design	Setting	Period of study	Male	Age	Sample size	Diabetes	Hypertension	Hyperlipidemia	Diagnosis	NOS
Toriguchi 2015 (13)	RCS	Japan	2002-2012	LLR: 13 OLR: 49	LLR: 64(43-76) OLR: 65(42-88)	LLR: 13 OLR: 69	LLR: 8 OLR: 11	LLR: 5 OLR: 15	LLR: 2 OLR: 8	Benign lesions, HCC, and metastatic liver tumors	6/9
Uchida 2016 (14)	RCS	Japan	2010-2015	LLR: 7 OLR: 10	LLR: 70.5(9.4) OLR: 67.1(6.2)	LLR: 12 OLR: 10	LLR: NA OLR: NA	LLR: NA OLR: NA	LLR: NA OLR: NA	Benign lesions, HCC, ICC, and metastatic liver tumors	7/9
Yu 2016 (10)	RCS	China	2013-2014	LLR: 6 OLR: 21	LLR: 49.4(12.7) OLR: 48.5(8.6)	LLR: 14 OLR: 51	LLR: NA OLR: NA	LLR: NA OLR: NA	LLR: NA OLR: NA	HCC and liver cavernous hemangioma	7/9
Ome 2019 (1)	RCS	Japan	2014-2017	LLR: 41 OLR: 54	LLR: 70(41-87) OLR: 67.5(25-83)	LLR: 79 OLR: 63	LLR: 23 OLR: 34	LLR: NA OLR: NA	LLR: NA OLR: NA	Benign lesions, HCC, ICC, and metastatic liver tumors	8/9
Heise 2021 (15)	RCS	Germany	2015-2019	LLR: 36 OLR: 45	LLR: 64.4(10.2) OLR: 64.5(12.3)	LLR: 68 OLR: 68	LLR: NA OLR: NA	LLR: NA OLR: NA	LLR: NA OLR: NA	HCC, ICC, and metastatic liver tumors	8/9
Inoue 2021 (16)	RCS	Japan	2010-2018	LLR: 18 OLR: 11	LLR: 66(29-82) OLR: 67(45-87)	LLR: 34 OLR: 18	LLR: 12 OLR: 6	LLR: NA OLR: NA	LLR: NA OLR: NA	Metastatic liver tumors	7/9
Ishihara 2021 (6)	RCS	Japan	2000-2019	LLR: 75 OLR: 158	LLR: NA OLR: NA	LLR: 111 OLR: 203	LLR: 42 OLR: 83	LLR: 62 OLR: 118	LLR: 29 OLR: 50	HCC	7/9
Yoon 2022 (9)	RCS, PSM	Korea	2009-2018	LLR: 78 OLR: 79	LLR: 56.38(10.26) OLR: 55.9(10.09)	LLR: 120 OLR: 120	LLR: 20 OLR: 20	LLR: NA OLR: NA	LLR: NA OLR: NA	Benign lesions, HCC, ICC, GIST, and metastatic liver tumors	9/9
Nakamura 2024 (17)	RCS	Japan	2021-2020	LLR: 21 OLR: 25	LLR: 68(45-82) OLR: 64(37-80)	LLR: 31 OLR: 32	LLR: NA OLR: NA	LLR: NA OLR: NA	LLR: NA OLR: NA	HCC, ICC, and metastatic liver tumors	6/9

HCC, hepatocellular carcinoma; ICC, intrahepatic cholangiocellular carcinoma; LLR, laparoscopic liver resection; NA, not available; OLR, open liver resection; PCS, prospective cohort study; PSM, propensity score matching; RCS, retrospective cohort study.

### 3.3 Meta-analysis

#### 3.3.1 Mortality

**Figure 2:**
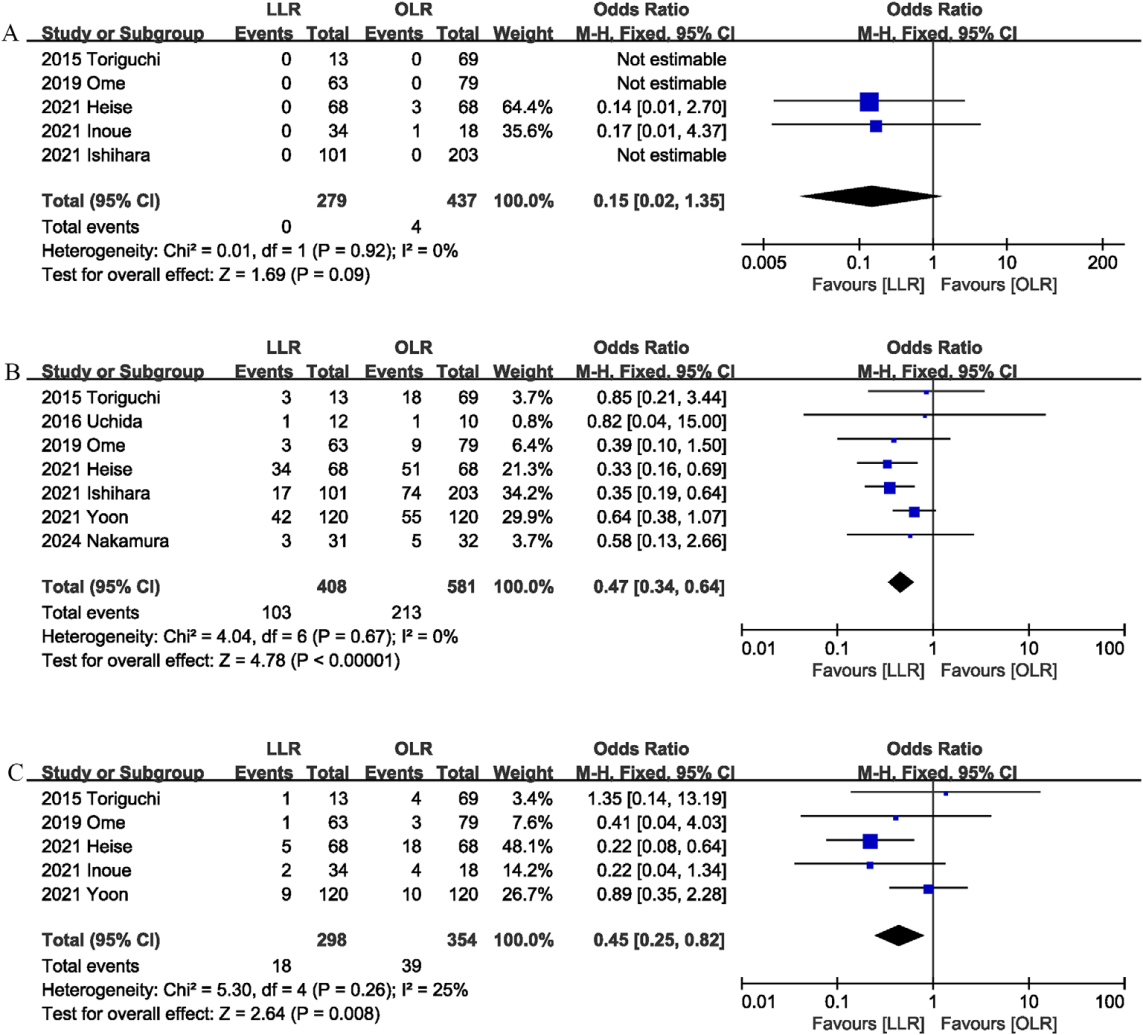
Comparison of primary outcomes between the two groups. **(A)** mortality, **(B)** overall morbidity, and **(C)** major complications.

Five studies (1, 6, 13, 15, 16) reported data on mortality. The combined results of the 5 studies showed that there was no significant difference between the LLR group and the OLR group regarding this outcome with low heterogeneity (OR 0.15, 95% CI 0.02, 1.35; Heterogeneity: I^2^ = 0%, P = 0.92) (**Figure 2A**) (**Table 3**).

**Table 3:** Summary of results from all outcomes.

Outcomes	No. of studies	Events for LLR	Events for OLR	Effect size	95%CI	P	I^2^ (%)
Overall complications	7	103/408	213/581	0.47	0.34, 0.64	<0.00001	0
Mortality	5	0/279	4/437	0.15	0.02, 1.35	0.09	0
Major complications	5	18/298	39/354	0.45	0.25, 0.82	0.008	25
Surgical site infection	6	4/310	38/469	0.18	0.07, 0.48	0.0006	0
Bile leak	6	10/310	29/469	0.45	0.22, 0.95	0.04	14
Liver failure	5	1/279	5/437	0.47	0.11, 2.01	0.31	25
Blood transfusion	4	12/264	57/336	0.15	0.02, 1.17	0.07	70
Blood loss	4	–	–	-329.12	-623.35, -34.88	0.03	84
Operation time	7	–	–	-35.75	-101.56, 30.05	0.29	92
Hospital stay	8	–	–	-5.20	-7.43, -2.97	<0.00001	67

#### 3.3.2 Morbidity

Seven studies (1, 6, 9, 13–15, 17) assessed overall complication. The pooled results suggested that LLR significantly reduced the overall complication rate (OR 0.47, 95% CI 0.34, 0.64, P < 0.00001), with low heterogeneity (I^2^ = 0%, P = 0.67) (**Figure 2B**).

#### 3.3.3 Major complications

Major complications (Clavien–Dindo ≥ 3) was evaluated in 5 studies (1, 9, 13, 15, 16), and the pooled results showed that LLR had lower major complications rate than OLR (OR 0.45, 95% CI 0.25, 0.82; heterogeneity: I^2^ = 25%, P = 0.26) (**Figure 2C**).

#### 3.3.4 Blood loss

**Figure 3:**
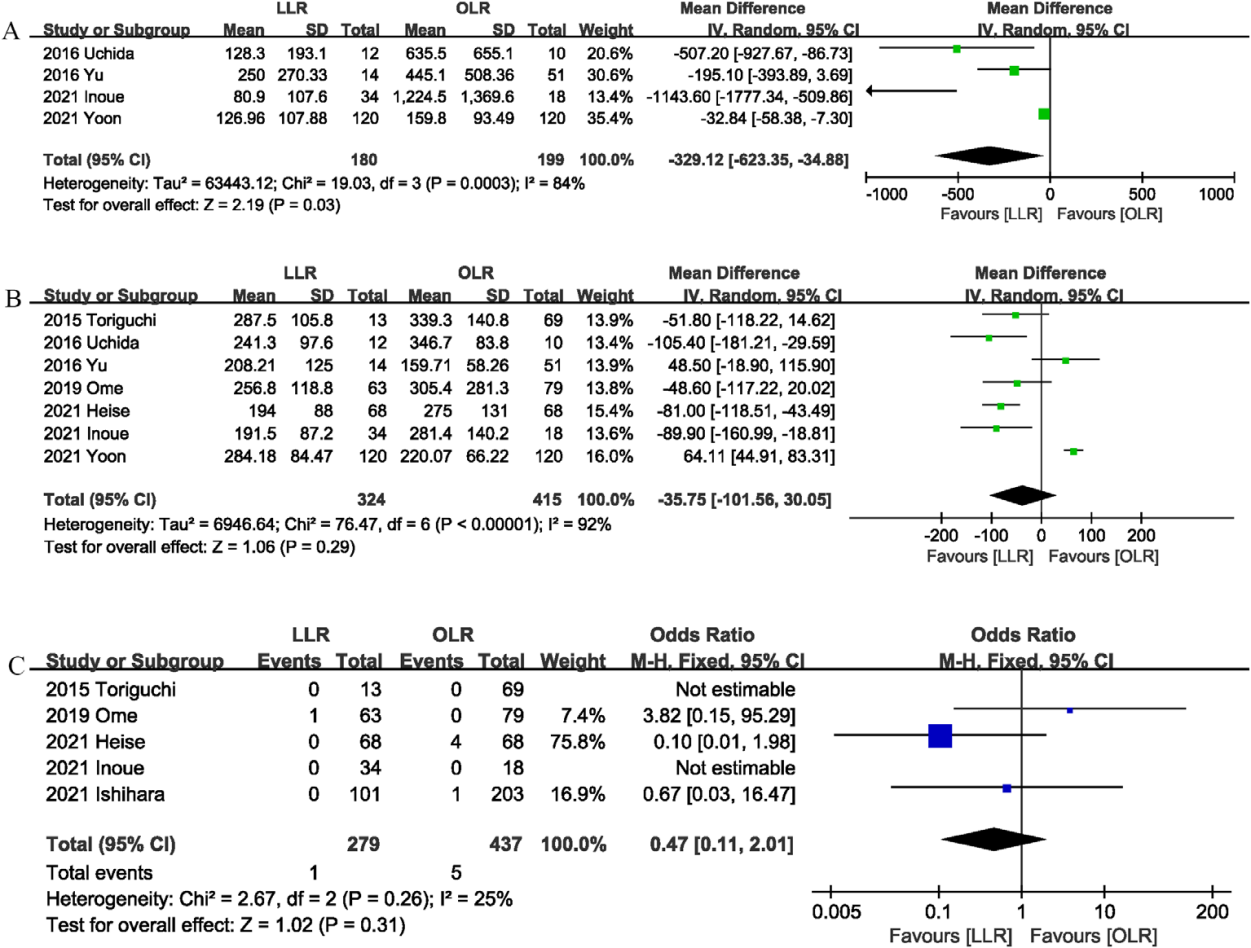
Comparison of secondary outcomes between the two groups. **(A)** intraoperation blood loss, **(B)** operation time, and **(C)** liver failure.

Four studies (9, 10, 14, 16) provided information on intraoperative blood loss. The combined results showed that LLR significantly reduced the blood loss (MD, -329.12 mL; 95% CI, -623.35, -34.88, P = 0.03; I^2^ = 84%) (**Figure 3A**).

#### 3.3.5 Operation time

The operation time was reported in 7 trials (1, 9, 10, 13–16). The combined results showed that the LLR group has similar operation time as compared with the OLR group (MD, -35.75 mins; 95% CI, -101.56, 30.05, P = 0.29) (**Figure 3B**).

#### 3.3.6 Liver failure

Liver failure was reported in 5 studies (1, 6, 13, 15, 16), and the combined effect size suggested that the liver failure rates were comparable between the two groups (OR 0.47, 95% CI 0.11, 2.01, P = 0.31; I^2^ = 25%) (**Figure 3C**).

#### 3.3.7 Blood transfusion

**Figure 4:**
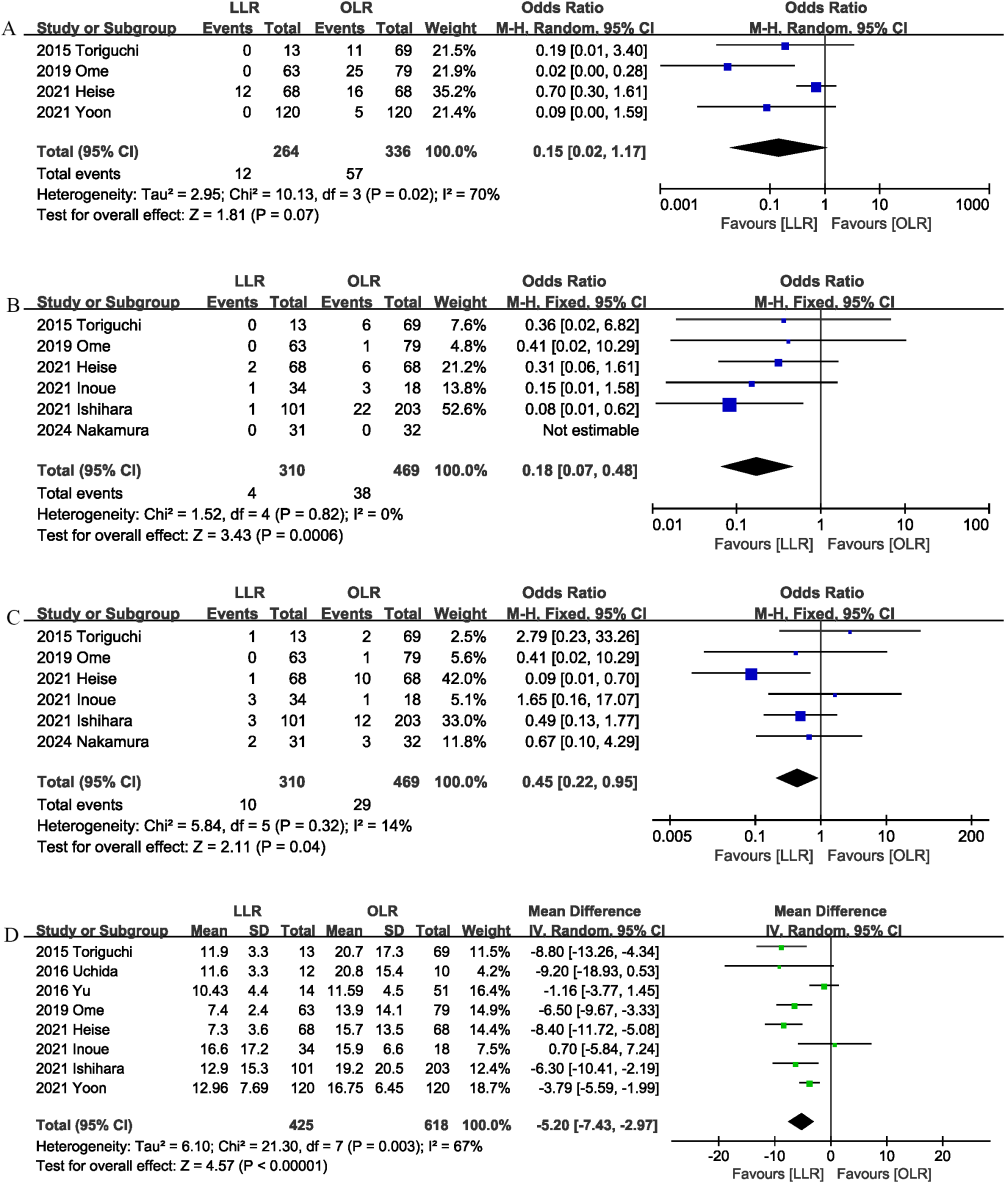
Comparison of secondary outcomes between the two groups. **(A)** blood transfusion, **(B)** surgical site infection, **(C)** bile leak, and **(D)** length of stay.

Four studies (1, 9, 13, 15) reported blood transfusion. No significant differences were observed between the two groups (OR 0.15, 95% CI 0.02, 1.17, P = 0.07), and heterogeneity was low (I^2^ = 70%, P = 0.02) (**Figure 4A**).

#### 3.3.8 Surgical site infection

Surgical site infection was evaluated in 6 studies (1, 6, 13, 15–17), and the pooled results showed that LLR had lower surgical site infection rate than OLR (OR 0.18, 95% CI 0.07, 0.48, P = 0.0006) (**Figure 4B**).

#### 3.3.9 Bile leak

Six studies (1, 6, 13, 15–17) compared bile leak rates between the LLR and OLR groups. The combined results showed that LLR effectively reduced the bile leak rate (OR 0.45, 95% CI 0.22, 0.95; heterogeneity: I^2^ = 14%, P = 0.32) (**Figure 4C**).

#### 3.3.10 Length of stay

The length of the hospital stay was reported in 8 studies (1, 6, 9, 10, 13–16). According to the results of this meta-analysis, the length of hospital stay was significantly shorter in the LLR group than in the OLR group (MD, -5.20 days; 95% CI, -7.43, -2.97, P < 0.00001) (**Figure 4D**).

### 3.4 Sensitivity analysis

Sensitivity analysis showed that no single study affected the overall effect size of the mortality, overall morbidity, operation time, surgical site infection, liver failure, or length of stay. The sensitivity analysis suggested that the total effect size of major complication changed significantly when the study by Heise et al. (15) (OR, 0.67; 95% CI, 0.32, 1.39, P = 0.28; I^2^ = 0%). The size of the pooled effect of the blood transfusion was influenced by Heise et al. (15) (OR, 0.06; 95% CI, 0.01, 0.33, P = 0.001; I^2^ = 0%). The size of the pooled effect of the bile leak was influenced by Ishihara et al. (6) (OR, 0.43; 95% CI, 0.18, 1.07, P = 0.07; I^2^ = 32%) or Heise et al. (15) (OR, 0.72; 95% CI, 0.31, 1.65, P = 0.43; I^2^ = 0%). The total effect size of the blood loss was influenced by Inoue et al. (16) (MD, -166.48 mL; 95% CI, -375.12, 42.16, P = 0.12), Yu et al. (10) (MD, -493.80 mL; 95% CI, -1090.89, 103.29, P = 0.11) or Uchida et al. (14) (MD, -275.70 mL; 95% CI, -594.37, 42.96, P = 0.09).

## 4 Discussion

To our knowledge, this is the first meta-analysis to evaluate the short-term outcomes of LLR versus OLR for liver tumors in obese patients. The pooled results of this study showed that, compared with OLR, LLR significantly reduced postoperative morbidity, major complications, surgical site infection and bile leak, intraoperative blood loss, and length of hospital stay. In addition, there were no significant differences between the two groups in postoperative mortality, liver failure, and operation time. These results have important clinical value. Because obesity is a widespread medical condition worldwide, it affects a large percentage of liver resection patients. Our study provides evidence to support the use of LLR in obese patients, and these results may help surgeons to provide a valuable reference when selecting the appropriate surgical approach for obese patients.

Postoperative complications not only prolong the length of hospital stay of patients, increase the cost of patients, but also affect the long-term survival of patients (18, 19). Matsuda et al. (19) found that postoperative complications significantly reduced 5-year overall survival and disease-free survival in patients undergoing liver resection. In obese patients, the increased thickness of the abdominal wall and the large amount of fat in the abdominal cavity add additional technical challenges during open surgery, limiting hand movement and visual range (6, 20). Obesity has been shown to be an important risk factor for increased morbidity after open surgery (6, 9, 21). Previous studies have shown that the total postoperative complications in obese patients undergoing OLR are as high as 41%, which is consistent with the results of our meta-analysis (7). In our included study, the overall complication rate after OLR was 36.2%, while laparoscopic surgery significantly reduced the overall complication rate (24.8%). In addition, our results suggest that laparoscopic surgery also reduces the risk of major complications. This may be due to the fact that in laparoscopic surgery, pneumoperitoneum and high-power microscopy (even deep in caudal view) can provide sufficient free space and a better field of view to perform the procedure. Similarly, several studies (22–24) have observed the benefit of laparoscopic surgery in reducing postoperative complications in obese patients in other abdominal procedures, such as appendectomy and colorectal surgery. In addition, our results showed that postoperative mortality was comparable in the laparoscopic and open surgery groups. Previous evidence suggests that obesity may have long-term metabolic and systemic effects on the body, such as metabolic disorders, increased risk of atrial fibrillation, cardiovascular disease, and overall mortality (25, 26). Therefore, the establishment of individualized perioperative management strategies, such as nutritional counseling to establish a healthy and balanced diet, physical exercise (26), and monitoring obesity-related diseases, may help improve surgical outcomes.

Previous studies have shown that the incidence of surgical site infection in obese patients is significantly higher than that in non-obese patients (6, 15, 22). This may be due to the association between obesity and impaired lymphocyte reactive immunity, inadequate collagen formation, and lack of blood vessels under the adipose tissue (27). Our results suggest that LLR can significantly reduce the incidence of postoperative surgical site infection. This is similar to the results of a meta-analysis by Shabanzadeh et al. (27), which included 8 RCTs and 36 observational studies, showing that laparoscopic surgery significantly reduced the risk of surgical site infection in obese patients undergoing bariatric surgery and non-bariatric surgery (OR = 0.19; 95% CI 0.08-0.45). This may be due to the fact that laparoscopic surgery induces a milder pro-inflammatory response than open surgery and better preserves postoperative immune function (28). In addition, the smaller surgical trauma of laparoscopic surgery may also be the reason for the lower incidence of surgical site infections (27).

Obesity is associated with increased blood loss and prolonged operation time during liver resection (6). Increased intraoperative blood loss may compromise a patient’s surgical outcome (29, 30). Laparoscopic surgery has the following advantages over open surgery and may help reduce intraoperative blood loss. On the one hand, laparoscopic surgery has a high pneumoperitoneum effect, which reduces the impact of obesity and leads to less blood loss than open surgery. On the other hand, compared with open surgery, laparoscopy can provide a clearer field of view, facilitate fine liver dissection, and reduce intraoperative blood loss (17). We also observed the benefit of laparoscopic surgery in reducing intraoperative blood loss, and the operation time was comparable to that of OLR. In addition, our results suggest that the laparoscopic surgery group had a shorter hospital stay than the open surgery group, which may be associated with lower postoperative complications and less intraoperative blood loss. LLR offers benefits such as reduced complications and faster postoperative recovery. However, the experience of the surgeon may affect the outcome of surgery. Complex LLR is technically demanding and requires a skilled surgeon to perform it. Previous studies have shown that the learning curve for LLR ranges from 15 to 60 cases (31). In addition, the gradual implementation of LLR combined with simulation-based training programs may reduce the impact of the learning curve on clinical outcomes (32).

Our study has several advantages. On the one hand, we conducted a comprehensive literature search that included a wide range of evidence, which increased the reliability of our results. On the other hand, the results of the sensitivity analysis confirm the robustness of our main results.

Our meta-analysis has the following limitations. First, the studies we included were all non-RCTs with potential for bias. Second, some outcome measures (such as blood transfusion rates) are based on data from a small number of studies, and more studies are needed to confirm them. Furthermore, liver resection encompasses various techniques, ranging from partial to major liver resection, with differing risks and levels of technical difficulty. Of the studies we included, five included patients who underwent different liver resection techniques, one included only patients who underwent major liver resection, and three did not describe the details of the liver resection. Therefore, whether LLR is superior to OLR in specific liver resection techniques needs further investigation. For outcomes with low heterogeneity, such as postoperative mortality and operation time, LLR demonstrates clear advantages over OLR with a certain degree of reliability. However, high heterogeneity was found in some outcome measures (intraoperation blood loss, operation time and transfusion rates), hindering the accurate estimation of the outcomes, and these results need to be treated with caution. In addition, comorbidities may affect the outcome of the study. Of the studies we included, only five provided information on comorbidities. Due to limited data, we were unable to further assess the specific impact of comorbidities on the results of the study. Future studies need to adequately balance these confounding factors, such as using PSM or randomized controlled designs. Finally, while BMI is the most widely used and simplest measure of obesity, other measures such as visceral fat measurement, waist and hip circumference, and waist-to-hip ratio may be relevant to surgical outcomes, and future studies need to further consider the impact of these measures on study results.

In conclusion, this meta-analysis, based on currently available evidence, our meta-analysis suggests that laparoscopic surgery is superior to open surgery in terms of intraoperation blood loss, postoperation complications, and length of hospital stay in obese patients. In addition, no significant differences were observed in terms of operation time and postoperation mortality. The benefits of laparoscopic surgery may make it a preferred option for obese patients. High-quality RCTs to validate the benefits of LLR are warranted.
